# Granulomatous fasciitis followed by morphea profunda: Is granulomatous fasciitis part of a spectrum of deep morphea? A case report and review of the literature

**DOI:** 10.1002/ccr3.1608

**Published:** 2018-06-04

**Authors:** Angie Christensen, Christina Di Loreto, Edward Smitaman, Taraneh Paravar, Karra A. Jones, Monica Guma

**Affiliations:** ^1^ Department of Rheumatology School of Medicine University of California, San Diego La Jolla CA USA; ^2^ Department of Pathology School of Medicine University of California, San Diego La Jolla CA USA; ^3^ Department of Radiology School of Medicine University of California, San Diego La Jolla CA USA; ^4^ Department of Dermatology School of Medicine University of California, San Diego La Jolla CA USA; ^5^ Department of Pathology University of Iowa Iowa City IA USA

**Keywords:** deep morphea, eosinophil fasciitis, granulomatous fasciitis, Lyme disease, morphea, morphea profunda

## Abstract

Although eosinophilic fasciitis is known to be part of the deep morphea spectrum, this first report of the coexistence of granulomatous fasciitis and morphea profunda suggests that granulomatous fasciitis may also be a part of the spectrum of deep morphea.

## 1 CASE REPORT

A 24‐year‐old Caucasian man presented with a several month history of muscle pain, fatigue and insidious onset of pitting edema to his lower extremities. His symptoms progressed to include bilateral arm swelling, muscle pain to the thenar eminence of both hands, and paresthesias to his hands. He did not have a rash, joint pain, Raynaud phenomenon, oral ulcers, fever, hardening of the skin or weight loss, and he denied any cardiac, respiratory, genitourinary, or gastrointestinal symptoms. Prior to symptom onset, he had traveled to South America and reported possible ingestion of undercooked meat as well as swimming in a river and lake. He had frequent visits to the Northeastern United States and had recently spent an extended period of time outdoors in Rhode Island. Medical, surgical, and family histories were unremarkable, and he was not taking any medications. He had a history of mild alcohol intake, no history of smoking, and some marijuana use. On examination, his vital signs revealed a blood pressure of 104/57 mm Hg, pulse of 56 bpm, and normal temperature. He had no significant findings on head, neck, cardiovascular, respiratory, or abdominal exam. He had no cervical, axillary, or inguinal lymphadenopathy. He had significant pitting edema on his feet and legs extending up to his knees as well as non‐pitting edema on the dorsum of both hands. Although there was edema, the skin was soft without any significant hardening and was without any overlying erythema. There were no signs of skin dimpling or grooves. His neurological examination revealed normal strength.

His initial laboratory work was significant for a mild eosinophilia of 700 with a normal white blood cell count, hemoglobin, and platelets. He had normal calcium, creatinine, and electrolyte levels. His alanine aminotransferase was slightly elevated and total protein slightly low, but he had normal albumin and bilirubin. His thyroid‐stimulating hormone was slightly increased, but his free T4 and total T3 were normal. His urinalysis was normal. He had a normal level of creatine phosphokinase, sedimentation rate, and C‐reactive protein. Further workup revealed a positive anti‐nuclear antibody with a titer of 1:160 with a speckled pattern. His extractable nuclear antigen panel was negative, including Scl‐70. His ANCA, myeloperoxidase and proteinase‐3 serum studies were also negative. His ACE level and complement levels were normal. Infectious workup revealed negative stool studies for culture, ova, and parasites. Antibodies for HIV, CMV, and Trichinella were negative. Serum for histoplasmosis, cryptosporidium, coccidiomycosis, and interferon gamma release assay were negative. A blood smear for parasites was negative. An ELISA IgG/IgM test for Lyme disease was positive with subsequent testing with Western blot strongly positive for IgG (eight out of ten bands positive) and also positive for IgM (two out of three bands positive).

**Figure 1:**
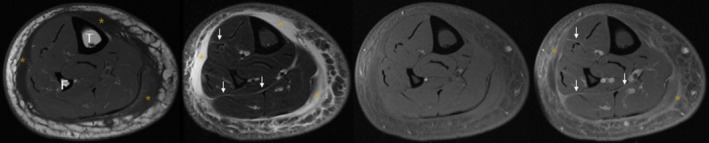
Initial magnetic resonance imaging of the right lower extremity (axial plane from left to right with TR/TE times [in msec]: T1 [564/12], T2 fat‐suppressed [4517/70], T1 precontrast fat‐suppressed [599/12], and T1 postcontrast fat‐suppressed [599/12]) demonstrates extensive circumferential edema and enhancement of the superficial soft tissues and superficial fascia (asterisks) and, to a lesser extent, the deep fascia (arrows)

**Figure 2:**
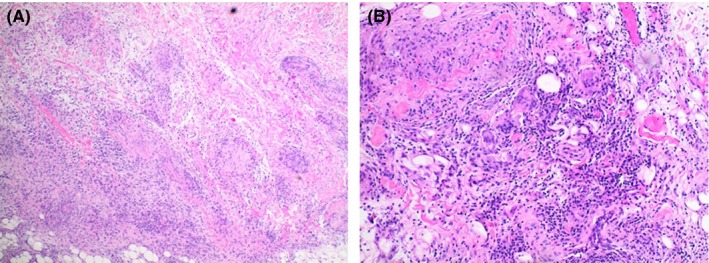
A and B, The fascia shows exuberant granulomatous inflammation. The inflammatory infiltrate is composed of enumerable histiocytes, many lymphocytes (predominantly CD3 positive T cells), rare plasma cells, and very rare eosinophils (hematoxylin‐eosin, magnification x40 [A] x100[B])

The patient had extensive imaging done with a normal CT scan of his neck, chest, abdomen, and pelvis. An echocardiogram was also normal. Due to a previous negative workup along with persistent pain and swelling, an MRI of his right lower extremity was performed (see Figure 1). Imaging demonstrated extensive circumferential edema with enhancement of the superficial soft tissues, superficial fascia, and, to a lesser extent, deep fascia of the lower leg. Taking into account the mild peripheral eosinophilia and the imaging findings, a diagnosis of eosinophilic fasciitis was considered. To complete the workup, a biopsy of the fascia, muscle, and adipose tissue of the left calf was taken (see Figure 2). The biopsy did not include the dermis. Surprisingly, there was no evidence of eosinophilic fasciitis. Instead, the specimen illustrated a striking granulomatous fasciitis and vasculitis. The fascia showed exuberant granulomatous inflammation (Figure 2A) with an inflammatory infiltrate that was made up predominately of histiocytes and CD3‐positive T cells with very rare eosinophils (Figure 2B). The granulomatous inflammation centered primarily on small‐ to medium‐sized blood vessels and was non‐necrotizing. While the vessels did not display overt fibrinoid necrosis, they did appear damaged with loss of endothelial cells confirmed with CD31 immunostaining. The inflammatory infiltrate was seen extending into adipose tissue and particularly around blood vessels within the fat. The adjacent skeletal muscle also showed perivascular inflammation and vasculitis in both the perimysial and endomysial compartments. There was no endomysial fibrosis, fatty infiltration, or inflammation surrounding muscle fibers. AFB (acid‐fast bacteria) and Wade‐Fite stain were negative for mycobacterial organisms. GMS (Grocott‐Gomori's methenamine silver) stain was negative for fungal organisms. Due to the positive Western blot for Lyme, a Warthin‐Starry silver nitrate stain was performed to evaluate for spirochetes; however, no definitive organisms were seen. A Borrelia PCR analysis of the tissue was performed as well, but no DNA was detected.

**Figure 3:**
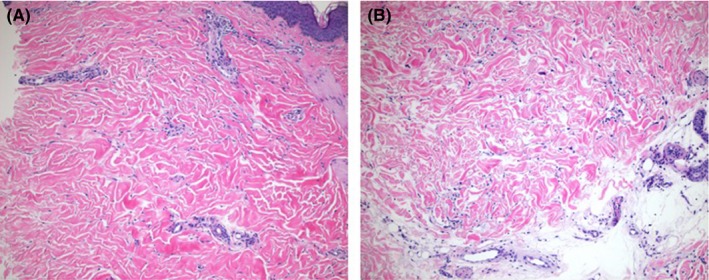
A and B, Sections show marked sclerosis with excessive collagen deposition in both mid‐dermis (A) and deep dermis (B) (hematoxylin‐eosin, magnification x100)

The patient was given a diagnosis of granulomatous fasciitis along with a diagnosis of Lyme disease. He was first treated with doxycycline for 42 days straight due to initial Lyme serology being positive. The calf biopsy revealing fasciitis was not performed until the patient was 3 weeks into the doxycycline course. Once fasciitis was diagnosed, he was started on a prednisone taper starting at 1 mg/kg/day for a week with taper by 10 mg every 2 weeks. After 2 months of treatment with prednisone, the patient had near resolution of symptoms. A repeat MRI was performed 82 days after the initial MRI with the previously seen changes consistent with fasciitis nearly completely resolved with only a thin sliver of edema over the superficial fascia. On re‐evaluation of the patient, he reported skin changes to his left upper arm at 20 mg of prednisone per day and skin changes to his left forearm at 7.5 mg per day. Examination of the upper arm revealed an atrophic patch with some overlying erythema and examination of the forearm showed indurated, bound‐down, tense skin with a slight groove and minimal overlying hyperpigmentation. The rest of the dermatological examination was within normal limits. Skin biopsy revealed marked septal thickening with sclerosis, sparse lymphoplasmacytic infiltrate along the dermal subcutaneous junction, and swollen, homogenized collagen fibers with diminished spaces between the fibers (see Figure 3). The clinical examination along with pathology revealing dermal and subcutaneous sclerosis was consistent with a diagnosis morphea profunda. Lyme serology was repeated, but Western blot for IgG was negative. A scleroderma antibody panel was negative as well. He was started on methotrexate and a higher dose of prednisone.

## 2 DISCUSSION

Upon review of the literature, granulomatous fasciitis is rarely reported. In 1982, Lewkonia, et al^1^ described two cases with clinical and histological features of eosinophilic fasciitis, but also with histologic findings of granuloma formation in association with prominent vasculitis. The first case described a 47‐year‐old man with myalgias, arthralgias, and edema with subcutaneous induration of the distal extremities, but no signs of cutaneous vasculitis. Laboratories revealed peripheral eosinophilia. Calf biopsy was consistent with granulomatous vasculitis, thickened fascia with an eosinophilic infiltrate, and granuloma formation in the fascia. The patient was treated with a 20‐mg prednisone taper with resolution of most symptoms by 1 month. The second case described a 14‐year‐old girl with subcutaneous swelling and induration of both legs with subsequent development of upper extremity brawny edema and stiffness. She had mild peripheral eosinophilia with calf biopsy revealing fascial thickening with lymphocytic infiltrate with scattered eosinophils and prominent perivascular mononuclear infiltration with granuloma formation in the fascia, muscle, and dermis. She required prednisone 100 mg on alternate days with improvement in symptoms by 8 months.

Few cases of granulomatous fasciitis have been reported due to sarcoidosis, but muscle was also involved.^2^, ^3^, ^4^, ^5^ The first known cases were reported in 1990, with accounts of two female patients with histopathological proven granulomatous myositis associated with a fasciitis.^2^ Another reported case revealed fasciitis via FDG‐PET in a patient with known pulmonary sarcoidosis who presented with bilateral calf and thigh stiffness; however, no biopsy was performed.^4^ The most recent case revealed changes suggestive of fasciitis via MRI of the abdomen, pelvis, and thighs in a patient with weight loss without swelling or skin changes. Biopsy of the thigh revealed fasciitis with non‐caseating granulomas.^5^

Upon further literature review of granulomatous fasciitis, few other associated reports were found. Richard Prayson reviewed 2985 muscle biopsy specimens over a twelve‐year time period and found only 12 cases (0.4%) with granulomatous myositis. All cases exhibited non‐necrotizing granulomas with infiltrates predominately made up of lymphocytes and plasma cells. Staining for acid‐fast bacilli and methenamine silver was negative. Of the 12 cases, 6 were diagnosed with sarcoidosis, 2 with vasculitis, 2 not otherwise specified, and 2 with insufficient information for diagnosis.^6^ Vaccination causing a histiocyte reaction should also be considered in the differential of granulomatous fasciitis. Vaccinations with aluminum oxyhydroxide have been shown to cause a long‐term persistence of alum‐loaded macrophages at the site or prior intra‐muscular injection, forming a histiocytic lesion called macrophagic myofasciitis. It can be differentiated from other types of fasciitis by a crystalline inclusion in the macrophages and lack of granuloma formation.^7^ In veterinarian literature review, cases of granulomatous fasciitis have also been described in birds 8 (due to *B procyonis*), cats,^9^ and eels 10 (due to novel Mycobacterium species).

Although suspected in our patient, but proven to be otherwise, eosinophilic fasciitis can present in a similar fashion as granulomatous fasciitis. Eosinophilic fasciitis (EF) is a rare disorder, first described by Shulman in 1974.^11^ It is characterized by limb or trunk erythema and edema with subsequent development of collagenous thickening of the subcutaneous fascia. Initial findings are a nonpitting edema that can progress to induration with puckering and a woody texture (peau d'orange).^1^, ^11^ Unlike scleroderma, the skin of the hands and feet is generally spared and it is not associated with Raynaud phenomenon. Another typical finding is the groove sign (indentation along the course of the superficial veins upon elevation of the affected limb). The majority of patients with EF have a peripheral blood eosinophilia, which is transient and does not correlate with disease severity. About half will have an elevated sedimentation rate and C‐reactive protein. It is also associated with hypergammaglobulinemia. Fascial inflammation can be suggested by MRI, FDG‐PET/CT, or ultrasound. The diagnosis is made by full thickness incisional biopsy of the skin and subcutaneous tissues down to the muscle surface. Early in the disease course, edema and infiltration with lymphocytes, plasma cells, histiocytes, and eosinophils of the deep fascia and lower subcutis is a typical feature on biopsy. The majority of patients have eosinophil infiltrates; however, this finding may be absent. As the disease progresses, the inflammatory infiltrates disappear and the fascia and subcutis become thickened and sclerotic. Thickening and inflammation can also be seen in or around muscles. Perivascular infiltrates of lymphocytes are almost always present. Initial treatment for EF consists of systemic glucocorticoids, usually at a dose of 1 mg/kg/day with dose reduction based on skin softening which can take weeks to months. Higher doses of glucocorticoids should be used if EF signs and symptoms do not improve and eosinophilia persists. No randomized trials have evaluated therapy for EF, but several different immunosuppressants (methotrexate, mycophenolate, azathioprine, infliximab, rituximab, hydroxychloroquine) have been used in steroid resistant patients or patients who relapse.^11^

Due to the positive Lyme serology in our patient, granulomatous fasciitis caused by Lyme disease was considered. However, we were unable to demonstrate spirochete or Borrelia DNA in the tissue. Upon review of the literature, there have been no case reports of granulomatous fasciitis associated with Lyme disease. However, a causal relationship of *Borrelia burgdorferi* infection with eosinophilic fasciitis (EF) was suggested in 1987.^12^ Since then, a handful of cases have been reported with the association due to findings of EF on biopsy with serologic tests positive for Lyme (some using ELISA alone, immunoblot alone or both). These cases were unable to demonstrate *Borrelia* organisms in the biopsies. The timeline of EF onset in these case reports occurred anywhere from a few months to a few years after tick bite without typical Lyme symptoms in most cases.^13^ In 1996, Granter et al^13^ identified four cases of diffuse fasciitis associated with peripheral eosinophilia in which spirochetal organisms were identified on biopsy. In one patient, multiple organisms were seen using a modified Dieterle silver stain, two patients had only a single unequivocal organism detected via silver stain, while the fourth patient had a negative silver stain, but organisms were identified using rabbit polyclonal antibodies against *B burgdorferi*. One patient with detection by silver stain was found to have no clinical or serological evidence of Lyme disease. They proposed that these results indicate that some cases of EF are an expression of Lyme disease and that *borrelial fasciitis* should describe these lesions. There has been another case reported by Hashimoto et al^14^ in which a patient had onset of EF 2 years after a tick bite with positive ELISA for *B burgdorferi*, negative Warthin‐Starry stain, but detection of *B burgdorferi* flagellin DNA in the skin sample using PCR.

Although eosinophilic fasciitis is known to be part of deep morphea spectrum, this is the first report of coexistence of granulomatous fasciitis and morphea profunda. Morphea is a type of localized scleroderma in which excessive collagen is deposited in the dermis, subcutaneous tissue, or both. It lacks the internal organ involvement that can be seen with systemic sclerosis. Deep morphea, or morphea profunda, is one of the more rare clinical variants of morphea and can extend into the fascia and muscle.^15^ Morphea has been associated with both eosinophilic fasciitis and Lyme disease.^16^, ^17^ The pathogenesis of morphea is poorly understood, but environmental factors such as radiation, trauma, or infection have been proposed as a trigger. *B. burgdorferi* has been implicated in the causation of morphea; however, the role it plays remains controversial.^16^ It is thought that the inflammatory and immune process elicited by the presence of the spirochete within the patient's tissues plays a role in the onset of morphea. In European series, antibodies to *B. burgdorferi* were detected in one‐third to one‐half of the morphea patients; however, serological studies in the United States have reported negative results. Direct detection by culture of the affected skin has had limited success in Europe. PCR studies have been positive in about one‐seventh of cases in Europe and Asia, but consistently negative in the United States.^16^ Thus, *B. burgdorferi* is not accepted as a causative agent in morphea. Of interest to our case, there is also an ongoing discussion as to whether EF is a variant of morphea. There have been cases in which morphea and EF coexist; however, the combination is considered to be rare.^17^ The first association was reported by Lakhanpal in which 15 of 52 patients with EF also had morphea.^18^ Several case reports with features of both diseases have been presented since then with suggestion that these diseases are a more closely related continuum rather than separate diseases.^17^

## 3 CONCLUSION

In our patient's case, despite findings of limb edema with mild peripheral eosinophilia, the biopsy lacked evidence for eosinophilic fasciitis and instead revealed granulomatous inflammation of the fascia and vasculitis. A skin specimen was not available at the time of granulomatous fasciitis diagnosis to assess for morphea, and there were no dermatological manifestations at that time. It was not until prednisone tapering that the patient developed skin changes consistent with morphea profunda on the arm. During initial workup, the patient had positive Lyme serology via ELISA and immunoblot. Microbial testing of the fascia and muscle tissue was negative for Lyme disease; however, testing was performed after the patient had been on doxycycline for 3 weeks, which could have decreased the yield. Although we cannot claim causality, it is possible that the immune response against the Borrelia spirochete triggered an autoimmune response and the patient's current manifestations. It is also important to have in mind the diagnosis of Lyme disease as a possible trigger in autoimmune connective tissue disorders. Skin trauma has been involved in morphea profunda, which could be another environmental trigger for this patient to develop another manifestation in the spectrum of morphea profunda/eosinophilic fasciitis. Although eosinophilic fasciitis is known to be part of the deep morphea spectrum, this first report of the coexistence of granulomatous fasciitis and morphea profunda suggests that granulomatous fasciitis may also be a part of the spectrum of deep morphea.

## CONFLICT OF INTEREST

The authors whose names are listed in this manuscript have no affiliations with or involvement in any organization or entity with any financial interest or nonfinancial interest in the subject matter or materials discussed in this manuscript. The authors have also declared that no conflict of interest exists.

## AUTHORSHIP

All authors were involved in drafting the article or revising it critically for important intellectual content, and all authors approved the final version to be published. MG: had full access to all of the clinical data and takes responsibility for the integrity of the data and the accuracy of the manuscript. AC and MG: performed review of the literature and case report summary. ES: performed MRI images, interpretation, and description. CDL and KJ: performed pathology images, interpretation, and description. TP: performed dermatology images, interpretation, and description.
